# A Cluster of *MYB* Transcription Factors Regulates Anthocyanin Biosynthesis in Carrot (*Daucus carota* L.) Root and Petiole

**DOI:** 10.3389/fpls.2018.01927

**Published:** 2019-01-14

**Authors:** Massimo Iorizzo, Pablo F. Cavagnaro, Hamed Bostan, Yunyang Zhao, Jianhui Zhang, Philipp W. Simon

**Affiliations:** ^1^Plants for Human Health Institute, North Carolina State University, Kannapolis, NC, United States; ^2^Department of Horticultural Science, North Carolina State University, Raleigh, NC, United States; ^3^National Scientific and Technical Research Council (CONICET), Buenos Aires, Argentina; ^4^Estación Experimental Agropecuaria La Consulta, Instituto Nacional de Tecnología Agropecuaria (INTA), Mendoza, Argentina; ^5^Facultad de Ciencias Agrarias, Universidad Nacional de Cuyo, Mendoza, Argentina; ^6^Department of Horticulture, University of Wisconsin–Madison, Madison, WI, United States; ^7^Vegetable Crops Research Unit, United States Department of Agriculture–Agricultural Research Service, Madison, WI, United States

**Keywords:** *Daucus carota* L., anthocyanin accumulation, root and petiole, regulation, fine mapping, transcriptome, candidate genes

## Abstract

Purple carrots can accumulate large quantities of anthocyanins in their roots and –in some genetic backgrounds- petioles, and therefore they represent an excellent dietary source of antioxidant phytonutrients. In a previous study, using linkage analysis in a carrot F_2_ mapping population segregating for root and petiole anthocyanin pigmentation, we identified a region in chromosome 3 with co-localized QTL for all anthocyanin pigments of the carrot root, whereas petiole pigmentation segregated as a single dominant gene and mapped to one of these “root pigmentation” regions conditioning anthocyanin biosynthesis. In the present study, we performed fine mapping combined with gene expression analyses (RNA-Seq and RT-qPCR) to identify candidate genes controlling anthocyanin pigmentation in the carrot root and petiole. Fine mapping was performed in four carrot populations with different genetic backgrounds and patterns of pigmentation. The regions controlling root and petiole pigmentation in chromosome 3 were delimited to 541 and 535 kb, respectively. Genome wide prediction of transcription factor families known to regulate the anthocyanin biosynthetic pathway coupled with orthologous and phylogenetic analyses enabled the identification of a cluster of six *MYB* transcription factors, denominated *DcMYB6* to *DcMYB11*, associated with the regulation of anthocyanin biosynthesis. No anthocyanin biosynthetic genes were present in this region. Comparative transcriptome analysis indicated that upregulation of *DcMYB7* was always associated with anthocyanin pigmentation in both root and petiole tissues, whereas *DcMYB11* was only upregulated with pigmentation in petioles. In the petiole, the level of expression of *DcMYB11* was higher than *DcMYB7*. *DcMYB6*, a gene previously suggested as a key regulator of carrot anthocyanin biosynthesis, was not consistently associated with pigmentation in either tissue. These results strongly suggest that *DcMYB7* is a candidate gene for root anthocyanin pigmentation in all the genetic backgrounds included in this study. *DcMYB11* is a candidate gene for petiole pigmentation in all the purple carrot sources in this study. Since *DcMYB7* is co-expressed with *DcMYB11* in purple petioles, the latter gene may act also as a co-regulator of anthocyanin pigmentation in the petioles. This study provides linkage-mapping and functional evidence for the candidacy of these genes for the regulation of carrot anthocyanin biosynthesis.

## Introduction

Anthocyanins are secondary metabolites that give color to different organs of many plant species. These water-soluble purple, red, and blue pigments serve various roles in the plant, including attraction of animals and insects for seed dispersal and pollination, protection against ultraviolet light, amelioration of different abiotic and biotic stresses, and participation in physiological processes such as leaf senescence (reviewed by Gould et al., 2009). Consumption of anthocyanin-rich fruits and vegetables is associated with lower incidence of chronic diseases, including cardiovascular disease, diabetes, arthritis, neurological disorders and some types of cancers (reviewed by Prior, 2004). Most of the health benefits associated with anthocyanin consumption are attributed to the antioxidant and anti-inflammatory properties of these pigments (He and Giusti, 2010).

Anthocyanins are also used as food colorants. The extent of anthocyanin glycosylation and acylation have a significant effect on their chemical stability, bioavailability, and biological activities (reviewed by Prior and Wu, 2006). Glycosylation and acylation increase anthocyanin chemical stability (Giusti and Wrolstad, 2003) and therefore their potential usefulness as food colorants, whereas non-acylated anthocyanins are generally more bioavailable than their acylated counterparts (Charron et al., 2009). Thus, the relative content of acylated and non-acylated anthocyanin forms is relevant for their potential utilization for either nutraceutical purposes (e.g., for fresh consumption of anthocyanins with high bioavailability) or as food colorants (i.e., chemically stable pigments that do not oxidize or decompose under normal conditions used for food storage).

Purple carrots can accumulate large quantities of anthocyanins in their roots (Mazza and Miniati, 1993; Montilla et al., 2011). Purple carrots accumulate almost exclusively derivatives of cyanidin, although pelargonidin and peonidin glycosides have also been reported in trace quantities in some genetic stocks (Kammerer et al., 2003). Five cyanidin-based anthocyanins have been identified in purple carrots; three acylated and two non-acylated. The root content of these five anthocyanin pigments vary across carrot genetic stocks (Mazza and Miniati, 1993; Montilla et al., 2011). Anthocyanin pigmentation also varies between root tissues, ranging from fully pigmented roots (i.e., purple color in the root phloem and xylem) to pigmentation only in the outer-most layer of the phloem.

Progress toward understanding the genetic control of anthocyanin pigmentation in purple carrot has been made. Two simply inherited genes conditioning root anthocyanin pigmentation, *P*_1_ and *P*_3_, have been described and mapped to chromosome 3 in different carrot genetic backgrounds (Simon, 1996; Vivek and Simon, 1999; Yildiz et al., 2013; Cavagnaro et al., 2014). *P*_1_ controls root pigmentation in the ‘B7262’ genetic background (Simon, 1996), a purple-rooted carrot with green petioles, whereas *P*_3_ conditions purple pigmentation in the roots and petioles of P9547 and PI652188, central Turkish and Chinese carrots, respectively, with purple roots and leaves (Cavagnaro et al., 2014). Comparative linkage mapping using segregating populations developed from crosses using these three purple-root sources as progenitors, demonstrated that *P*_1_ and *P*_3_ correspond to different loci that map to chromosome 3 at more than 30 cM apart (Cavagnaro et al., 2014). In addition to *P*_1_ and *P*_3_, a simply inherited dominant locus conditioning purple pigmentation in the nodes, *P*_2_, has been described but not mapped (Simon, 1996).

Quantitative trait loci (QTL) for four individual root anthocyanin pigments and for total root anthocyanins, were genetically mapped, revealing co-localization of the QTL with the region of *P*_3_, further confirming that this region conditions root pigmentation in the P9547 background (Cavagnaro et al., 2014).

Currently, no candidate genes have been identified for *P*_1_ and *P*_3_. Structural anthocyanin genes were mapped in the B7262 genetic background, but none of them co-localized with *P*_1_, indicating that they are not controlling this trait, despite the fact that some genes were differentially expressed in purple *versus* non-purple roots (Yildiz et al., 2013). Similarly, differential gene expression of a MYB transcription factor, named *DcMYB6*, in purple and non-purple carrot roots was recently reported (Xu et al., 2017). Because of the lack of linkage or physical localization data for this gene relative to *P*_1_ and *P*_3_, its association with these loci is not possible.

In the present study, we performed high-resolution mapping in the *P*_3_ region combined with gene expression analyses (RNA-Seq) to identify candidate genes controlling anthocyanin pigmentation in the carrot root and petiole. Among our findings, we report on the identification and differential gene expression profile of a cluster of six anthocyanin related R2R3-MYB transcription factors in a ∼0.5 Mbp region associated with *P*_3_.

## Materials and Methods

### Plant Materials

Inheritance of purple pigmentation was studied in five segregating populations (two F_2_s, two F_3_s, and one F_5_) for a total of 1,997 phenotyped plants. Population 70349 was an F_2_ family (*N* = 497) derived from an initial cross between P4201 and B6320. P4201 is an inbred line with purple outer phloem and yellow xylem storage roots and purple leaves that was derived from a cross between inbred P9547, with purple xylem and phloem root color derived from Central Anatolia (Turkey), and B2566, an inbred with orange root color from diverse European sources. B6320 is an inbred with orange roots and green petioles derived from the European open-pollinated cultivars Nantes and Camberly. This population was previously characterized by Cavagnaro et al. (2014) for genetic mapping studies. Growing conditions and phenotyping for this population were previously described by Cavagnaro et al. (2014). Populations 5392 (*N* = 150) and 5394 (*N* = 171) are F_3_ families derived from self-pollination of two 70349 F_2_ purple plants.

**FIGURE 1:**
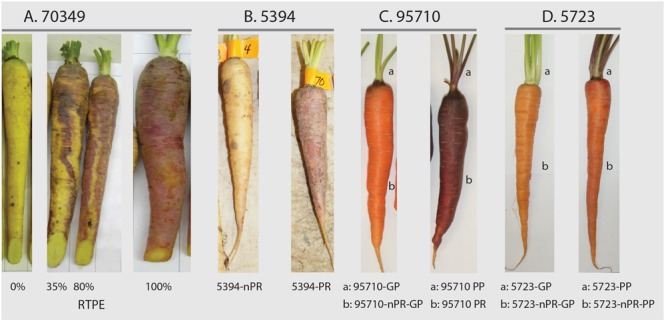
Carrot root and leaf petiole phenotypes in mapping populations 70349, 5394, 95710, and 5723. In 70349, the percentage of purple pigmentation covering the root surface (called “RTPE” for “root total pigment estimate”), was scored visually as described by Cavagnaro et al. (2014) and varied from 0 to 100% **(A)**. In 5394, root anthocyanin pigmentation was phenotyped based on the presence (5394-PR) or absence (5394-nPR) of purple pigment **(B)**. In 95710, petiole(a) and root(b) pigmentation was scored based on the presence (95710-PR, 95710-PP) or absence (95710-GP, 95710nPR-GP) of purple pigment **(C)**. In 5723, petiole(a) and root(b) pigmentation was scored based on the presence (5723-PP) or absence (5723nPR-GP, 5723nPR-PP, and 5723-GP) of purple pigment **(D)**. Additional phenotyping was performed in 5723 population, which segregated for green (GP), pale purple (pPP) and dark purple (dPP) petioles (Supplementary Figure S1).

Population 95710 (*N* = 668) was an F_2_ family derived from a cross between BP85682 and BP85683. BP85682 is a purple rooted carrot with purple leaves derived from a cross between a purple carrot from Homs, Syria, and the ultimate source of purple in 95710, and an orange-rooted carrot with green leaves (derived from diverse South American sources). BP85683 is an orange-rooted carrot with green leaves derived from diverse European sources. Population 5723 (*N* = 511) was an F_5_ family derived from an initial cross between a purple rooted carrot with purple leaves derived from an intercross between PI652188 (a purple carrot from China, and the ultimate purple source in 5723) and PI326011; and an orange-rooted carrot with green leaves derived from diverse European and South American sources. In addition to their phenotypic variation, these sources of anthocyanin pigmentation have different geographical origins, namely Turkey (P9547), China (BP85682), and Syria (BP85682). Root and petiole characteristics of the carrot segregating populations are shown in Figure 1 and Supplementary Figure S1.

In addition to the 70349 population previously characterized (Cavagnaro et al., 2014), seeds from the 5392, 5394, 5723, and 95710 populations were sown at the experimental field of the University of California Desert Research and Extension Center (Holtville, CA, United States) in the growing season of 2013–2014. A subset of 167 plants from the 95710 population was grown in 2014 at the University of Wisconsin Hancock Agricultural Research Station (Hancock, WI, United States). In both locations, plants were grown using conventional agricultural practices for carrot.

### Phenotyping and Segregation Analysis

Phenotyping for purple color in all five carrot populations was performed using a qualitative assessment, considering the presence or absence of purple pigmentation in carrot roots and/or petioles, as described by Simon (1996) (Figure 1). In addition, for population 5723, petioles were phenotyped as either ‘pale purple petiole’ (pPP), ‘dark purple petiole’ (dPP), or ‘green petiole’ (GP) (Supplementary Figure S1). Phenotypic data from each population were analyzed using the χ^2^ method to test the ratios for root and/or petiole anthocyanin pigmentation.

The anthocyanin pigmentation pattern of the purple roots from the different segregating populations varied clearly. Purple roots of 95710 accumulated anthocyanins in the outer-most cell layers of the root phloem, but not in the inner-phloem or xylem tissues (see Supplementary Figure S1). The same phenotype was expressed in population 2170, previously evaluated by Cavagnaro et al. (2014). In populations 5392, 5394, and 70349, root purple color followed an uneven blotchy pattern of pigmentation (see Supplementary Figure S1), with varying levels of purple pigmentation observed across the outer phloem, inner phloem and xylem. Whenever present, the extent of purple pigmentation in the inner phloem and xylem was usually less than on the surface of the root and the outermost phloem cell layers, suggesting variation in the expressivity of the trait in the different root tissues.

### Quantitative Root Color Phenotyping and HPLC Analysis in Population 70349

In order to explore in more detail the previously mapped region of carrot chromosome 3 harboring the major QTL controlling root total pigment content (named *RTPE-Q1*) and other four co-localized QTL for anthocyanin pigment content in the 70349 map (Cavagnaro et al., 2014), 234 additional individuals from the same F_2_ population were phenotyped and used for fine mapping, totaling 421 individuals. Two methods of phenotyping for anthocyanin expression in the roots were used. The first method was qualitative and rated the presence or absence of the purple pigment in the roots. The second method was also visually scored, using a rating scale of 0–100, which estimated the percentage of purple pigmentation covering the root surface, which estimates the root total pigment content in the 70349 background as previously described by Cavagnaro et al. (2014) (Supplementary Figure S1). In addition, tissue samples containing visible purple pigmentation were obtained from roots of the 70349 population. The samples were lyophilized and anthocyanins were extracted with acidified methanol, followed by high-performance liquid chromatography (HPLC) analysis of anthocyanin pigments as described by Kurilich et al. (2005). Supplementary Table S1 lists the five major carrot anthocyanin pigments (all cyanidin derivatives) identified and quantified in the present study. The data were expressed as percentage concentration of a given pigment relative to the total anthocyanin content, which derived from the sum of the content of the five individual anthocyanin compounds, as described previously (Cavagnaro et al., 2014) (Figure 1).

### Fine Mapping of QTL Conditioning Root Anthocyanin Pigmentation in 70349

Quantitative (HPLC and RTPE) phenotypic data and genotypic scores for 15 SNP markers in the *RTPE-Q1* map region of chromosome 3 were obtained for 421 individuals (234 were genotyped in the present study and 187 were previously genotyped by Cavagnaro et al., 2014) and were used for linkage map construction and QTL analysis. In addition, a new SNP marker located in the coding region of *DcMYB6*, an anthocyanin related R2R3-MYB recently proposed as a candidate gene controlling anthocyanin accumulation in carrot root (Xu et al., 2017), was developed and used to genotype individuals from the 70349 population. *DcMYB6* corresponds to carrot gene prediction DCAR000385 and is located in contig C10735702 (length = 3,207 bp) which was not anchored to chromosomes in the published carrot genome assembly (Iorizzo et al., 2016). Total genomic DNA of individual plants was isolated from lyophilized leaves following the protocol described by Murray and Thompson (1980) and quantified using Quant-iT^TM^ PicoGreen^®^ (Invitrogen, Paisley, United Kingdom). Genotyping was performed using KASPar Chemistry^1^ as previously described by Iorizzo et al. (2013). Primers used for mapping are listed in Supplementary Tables S2, S3. SNP scores for the 16 markers were converted into genotype codes using the A/H/B system for co-dominant markers segregating in an F_2_ population. JoinMap 4.0 software (Van Ooijen, 2006) was used for mapping, as previously described (Cavagnaro et al., 2014). QTL analysis was performed using R/qtl with the multiple imputations method (Broman and Sen, 2009). QTL detection included preliminary QTL identification using ‘scanone’ followed by QTL modeling. The largest LOD peak from the analysis was added to the QTL model and if the QTL model was significant, it was retained. This process was then repeated using ‘addqtl’, instead of ‘scanone’, followed by QTL modeling and testing for interactions until adding additional QTL to the model was no longer significant. The support intervals were calculated using a 1.5 LOD drop.

### Mapping of *P*_3_ in Diverse Carrot Genetic Backgrounds

In order to fine map the *P*_3_ locus, which is associated with root and petiole pigmentation in the 70349 and 2170 backgrounds (Cavagnaro et al., 2014) and co-localizes with *RTPE-Q1* in 70349, this trait was genetically mapped in 95710 (*N* = 501), 5394 (*N* = 171), and 5723 (*N* = 511) mapping populations, using SNP markers tightly linked to *P*_3_ and *RTPE-Q1*. In total, the fine mapping of *P*_3_ included 1,416 individuals from the three mapping populations described above. For selection of the SNP markers tightly linked to *RTPE-Q1* and *P*_3_, the sequences of the markers covering the RTPE QTL support interval in the 70349 high-resolution map were aligned against the carrot genome assembly (Iorizzo et al., 2016) and the corresponding region of the genome sequence was used to design primers and identify SNPs in the 95710, 5394, and 5723 populations.

Total genomic DNA of individual plants from 5394, 5723, and 95710 was isolated and evaluated as described above. Primers were designed using Primer3 (Koressaar and Remm, 2007; Untergasser et al., 2012) with the following parameters: end stability: 250; optimum Tm: 55°C; minimum size: 120 nt; maximum size: 1600 nt. In total, 86 primer pairs were synthesized (Supplementary Table S2). DNA from twenty genotypes with contrasting phenotypes (purple and non-purple) from each mapping population (5394, 5723, and 95710) were evaluated by PCR and Sanger sequencing as described by Iorizzo et al. (2011). To identify intra-population SNPs, amplicon sequences from each subset/population were analyzed using Sequencher software version 4.8 (GeneCodes Corporation, Ann Arbor, MI, United States). SNPs were identified for 20 amplicons and the sequences flanking each SNPs were used to set up the KASPar Chemistry assay and genotype the full set of progeny in each population, namely 5394 (*N* = 171), 5723 (*N* = 511), and 95710 (*N* = 501) (Supplementary Table S3).

### Delimitation of the Genomic Region in Chromosome 3 Harboring the *RTPE-Q1* and *P*_3_ Loci

In order to delimit and further analyze the genomic region controlling *RTPE-Q1* and *P*_3_, the sequences corresponding to the SNP markers located within *RTPE-Q1* support interval plus the immediately adjacent markers flanking *RTPE-Q1* support interval (in population 70349), and the SNP markers flanking the *P*_3_ locus in the 5394, 5723 and 95710 linkage maps, were aligned to the carrot genome assembly (Iorizzo et al., 2016) to determine the physical distance, in terms of number of nucleotides, amongst them. After this, SNP genotypic scores for these markers in all of the individuals in each population were used to establish haplotype blocks and to identify recessive (*aa*) to dominant (*A_*) recombinants (crossover point) spanning this region. Additional markers were then developed within this genomic region and used for additional genotyping in the mapping populations to identify new recombinants to further delimit the recombination breakpoints and therefore narrow the genomic region harboring the gene controlling these traits. For this purpose, 16 primer pairs were designed and used for PCR amplifications, followed by sequencing of the PCR products to identify sequence polymorphisms (SNPs or indels) as described above (Supplementary Table S2).

### Identification and Analysis of Candidate Genes for Root and Petiole Phenotypes in the Genomic Region Including *RTPE-Q1* and *P*_3_

To identify candidate genes controlling the *RTPE-Q1* and *P*_3_ loci, the sequences of the markers delimiting the recombination breakpoints in each mapping population were aligned against the carrot genome assembly and these regions were analyzed in search of structural and regulatory genes involved in the flavonoid and anthocyanin biosynthetic pathways.

For this purpose, coordinates of structural genes involved in the flavonoid and anthocyanin biosynthetic pathways were retrieved from the carrot genome annotation (manually curated by Iorizzo et al., 2016). To identify regulatory genes, PlantTFcat (Dai et al., 2013) was used to predict transcription factors (TFs) in carrot and 53 other plant genomes (Supplementary Table S4). Genes predicted as MYB-HB-like, bHLH, WD40-like and MYB/SANT, were extracted and used for orthologous and phylogenetic analysis since members of these four TF families are well known to control the flavonoid biosynthetic pathway in several species. Carrot TFs located in the fine mapped regions and that clustered together with anthocyanin-related TFs from other species in these analyses were considered candidate genes for *RTPE-Q1* and *P*_3_.

Orthologous analysis was performed using the OrthoMCL pipeline (Li et al., 2003) with an inflation value (-I) of 1.5. Phylogenetic analysis was conducted using MEGA version 7 (Kumar et al., 2016). In addition, carrot transcription factors associated with anthocyanin pigmentation in previous studies were aligned against the carrot genome assembly, and their coordinates were analyzed to determine if they were located within the *RTPE-Q1* region or nearby *P*_3_. Lists of plant genomes and genes used for these analyses are presented in Supplementary Tables S4–S7.

### Transcriptome Analysis

Eight tissue types representing different root and petiole phenotypes associated with the *RTPE-Q1* and *P*_3_ loci were sampled from 12-week old plants for total RNA and used for comparative transcriptome analyses. The following tissues were included: (1) purple root tissue from 5394 plants (5394-PR); (2) non-purple root tissue from 5394 (5394-nPR); (3) non-purple root tissue from 5723 plants with purple petioles (5723-nPR-PP); (4) dark purple petiole tissue from 5723 plants with orange roots (same #3 plants, 5723-PP); (5) non-purple root tissue from 5723 plants with green petioles (5723-nPR-GP); (6) green petioles tissue from 5723 plants with orange roots (same #5 plants, 5723-GP); (7) purple root tissue from 95710 plants with purple petioles (95710-PR); (8) purple petiole tissue from 95710 plants with purple root (same #7 plants, 95710-PP). Three biological replicates (i.e., roots or petioles from three plants) were sampled for each phenotype. Details on the different tissue/phenotype comparisons performed and the RNA-Seq data sets are presented in Supplementary Table S8.

Total RNA was extracted using the RNeasy Mini Kit (Qiagen, Valencia, CA, United States) in accordance with the manufacturer’s protocol. Contaminating DNA was removed with the TurboDNA-free kit (Life Technologies, Carlsbad, CA, United States). RNA quantity and integrity was confirmed with an Experion RNA StdSens Analysis kit (Bio-Rad, Hercules, CA, United States). All samples had RQI values >8.0.

For each sample, a single-end reads library was prepared at the Genomic Science Laboratory, North Carolina State University, Raleigh, NC, United States. Libraries were sequenced on Illumina HiSeq2500 lanes using 1 × 100-nt reads. Each library was sequenced in three independent lanes, representing three technical replicates.

Reads were filtered with Trimmomatic (Bolger et al., 2014), considering TruSeq adapters 2:30:10 LEADING:3 TRAILING:3 SLIDINGWINDOW:4:15 MINLEN:36. In this process, adapter sequences were removed from the reads and low-quality bases were trimmed from the 3′ end of the reads. The quality check of the remaining sequences was performed using FastQC (Andrews, 2010). High-quality short reads from each replicate were independently mapped against the carrot genome sequence (GenBank accession LNRQ01000000.1) using STAR version 020201 (Dobin et al., 2013) considering the following parameters: –alignEndsType = EndToEnd; –outFilterMismatchNmax = 2; –outFilterMultimapNmax = 20. Reads for each gene available from the V1.0 gene annotation of the carrot genome (Iorizzo et al., 2016) were quantified with the featureCounts standalone package (Liao et al., 2014), using only reads that mapped uniquely to the genome.

Total reads abundance for each gene was calculated by combining the read counts for a given locus from each technical replicate representing the expression level of that gene in each biological replicate per sample. Estimation of variance-mean dependence in count data and the test for the differential expressed genes (DEGs) was based on a model using the negative binomial distribution implemented in the DRSeq package (Anders and Huber, 2012) and considering FDR ≤ 0.05. Pearson correlations values between samples were calculated between technical replicates and samples 5723-nPR3(GP), 96710-PP3 and 5394-nPR3 were discarded due to high correlation with non-corresponding replicates.

### Real-Time Quantitative Reverse Transcriptase PCR

Twelve-week old plants were used to collect RNA samples. Total RNA was extracted from the same eight samples and tissues described above for transcriptome analysis, using three biological replicates for each tissue type. cDNA was synthesized by Invitrogen^TM^ SuperScript^TM^ III First-Strand Synthesis System (Thermo Fisher Scientific, MA) and diluted 10-fold for Reverse Transcription quantitative PCR (RT-qPCR) analyses. Reactions of RT-qPCR were carried out using the Power SYBR Green PCR Master Mix (Applied Biosystems) in an ABI 7500 Sequence Detection System by denaturation the DNA at 95°C for 10 min, followed by 40 cycles at 95°C for 15 s and 60°C for 40 s. Melting curve analyses were performed to verify the amplification specificity. Information of the primers used for RT-qPCR analysis is provided in Supplementary Table S9. For each gene and biological replicate, the experiments were carried out in triplicate. Relative quantification of gene expression was performed according to the ^2-ΔΔCT^ method described by Livak and Schmittgen (2001). EF-1α and ACTIN genes were used as internal controls to normalize the variability in expression levels (Tian et al., 2015). Using this method, we obtained the fold changes in gene expression normalized to internal control genes. All the experimental data were compared statistically and through one-way analysis of variance (ANOVA) using the software Statistical Product and Service Solutions (SPSS) v 23 (IBM, NY) followed by Tukey’s HSD test to determine the significant difference results.

## Results

### Inheritance of Purple Root and Petiole Color

In the F_2_ 70349 population and its F_3_ derivative, 5392, root purple pigmentation revealed a good fit to a 9:7 segregation ratio (χ^2^ = 0.003–2.02, *p* = 0.16–0.96) and both populations deviated significantly (*p* < 0.001) from the 3:1 ratio reported previously for *P*_1_ in other carrot backgrounds (Simon, 1996; Vivek and Simon, 1999; Yildiz et al., 2013), whereas segregation in 5394, another F_3_ derivative of 70349, fit a 3:1 ratio (χ^2^ = 0.70, *p* = 0.40) (Table 1). These data are consistent with previous results of Cavagnaro et al. (2014), reporting segregation ratios of 9:7 and 3:1 in other 70349-derived F_3_ and F_4_ populations developed from self-pollinating single purple-rooted plants, suggesting that two dominant loci interact epistatically in the genetic control of root purple pigmentation in the 70349 background. In the 95710 population, developed from a Syrian purple-carrot source, segregation ratio fit a 3:1 ratio in both growing locations evaluated (χ^2^ = 0.88–1.23, *p* = 0.27–0.35), suggesting a single dominant locus conditioning root purple pigmentation in this genetic background. The F_5_ population, 5723, had no purple roots, and segregated only for petiole pigmentation.

**Table 1:** Segregation of purple pigmentation in the roots and leaf petioles of six carrot populations.

Source of purple root and leaves (origin)	Population –generation – cultivation site^†^	Root/leaf phenotype of parent selfed plant^ζ^	Root						Petiole						
	
			Number of progeny			Expected ratio^β^	χ^2^	*p*-value	Number of progeny				Expected ratio^β^	χ^2^	*p*-value
			Purple	Non-purple	Total				Dark purple	Pale purple	Green	Total			
P9547 (Turkey)	70349-F_2_-WI	P/P	279	218	497	3:1	94.3	<0.001	323	–	90	413	**3:1**	2.27	0.13
						**9:7**	0.003	0.96					9:7	80.9	<0.001
	5392-F_3_-CA	P/P	93	57	150	3:1	13.5	<0.001	nd	nd	nd	nd	–	–	–
						**9:7**	2.02	0.16					–	–	–
	5394-F_3_-CA	P/P	133	38	171	**3:1**	0.70	0.40	nd	nd	nd	nd	–	–	–
						9:7	32.2	<0.001					–	–	–
BP85682 (Syria)	95710-F_2_-CA	P/P	365	136	501	**3:1**	1.23	0.27	365	nd	136	501	**3:1**	1.23	0.27
						9:7	56.1	<0.001					9:7	56.1	<0.001
	95710-F_2_-WI	P/P	120	47	167	**3:1**	0.88	0.35	120	nd	47	167	**3:1**	0.88	0.35
						9:7	16.5	<0.001					9:7	16.5	<0.001
PI652188 (China)	5723-F_5_-CA	NP/P	0	511	511	0:1	0.00	1.00	388		123	511	**3:1**	0.24	0.63
													9:7	80.4	<0.001
									115	251	123	123	**1:2:1**	0.61	0.74

*^†^Population name followed by generation and cultivation site, where CA and WI denote ‘Holtville, California’ and ‘Hancock, Wisconsin’, respectively. ^ζ^Root and petiole phenotypes were either purple (P) or non-purple (NP). ^β^Segregation ratios of 3:1 (single dominant gene) and 9:7 (two dominant genes with epistatic interaction) were considered and examined with χ^*2*^ tests. Genetic models with a good fit to the observed data are denoted in bold. No phenotypic data (nd) were available for petioles of populations 5392 and 5394*.

In all the populations and genetic backgrounds evaluated in this study, pigmentation in the petioles segregated as a simply inherited trait with purple being dominant over green (χ^2^ = 0.24–1.23, *p* = 0.27–0.63) (Table 1). These results are in agreement with the 3:1 segregation ratio (χ^2^ = 2.28, *p* = 0.13) reported previously for petiole purple color in the 70349 F_2_ population (Cavagnaro et al., 2014). Although a statistically sound 3:1 (purple: non-purple) ratio was found for petiole pigmentation in the populations evaluated in the present and previous studies, variation in the intensity of purple color was noted in the petioles of population 5723 (Supplementary Figure S1). Such variation of the purple color was not observed in other populations. The *post hoc* segregation analysis in population 5723, using three phenotypic classes [green (GP), pale purple (pPP), dark purple (dPP)], revealed a good fit of the observed data to a 1:2:1 ratio for GP:pPP:dPP (χ^2^ = 0.61, *p* = 0.74), suggesting an incomplete or partial dominance for purple petiole pigmentation in the 5723 background (Table 1).

### Fine Mapping of QTL Conditioning Root Purple Pigmentation in Population 70349

**FIGURE 2:**
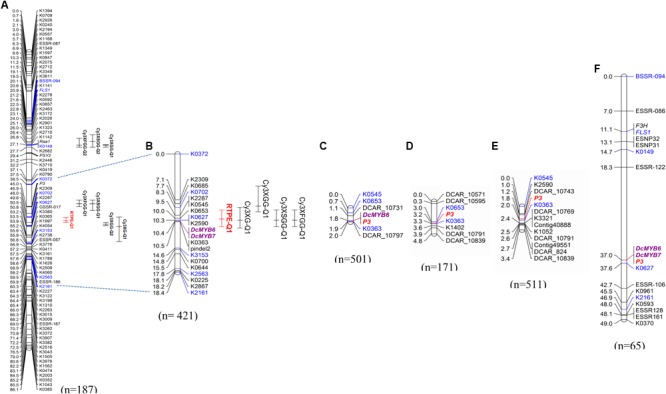
Genetic mapping of anthocyanin pigment traits in chromosome 3 of different carrot populations. QTL for ‘root total pigment estimate’ (RTPE) and root anthocyanin pigments (Cy3XG, Cy3XGG, Cy3XSGG, and Cy3XFGG) mapped in the original framework linkage map published by Cavagnaro et al. (2014) **(A)**. High-resolution mapping in the *RTPE-Q1* region using a larger 70349 population **(B)**. The connecting dotted lines indicate the flanking markers of the map region further analyzed in this work. In **(A,B)**, bars to the right of the linkage groups represent support intervals of the QTL. *RTPE-Q1*, explaining 52.3% of the variation, is denoted in red. The *P*_3_ locus, conditioning root and leaf pigmentation, was mapped in populations 95710 **(C)**, 5394 **(D)**, 5723 **(E)**, and 2170 **(F)**. Population size is indicated in parenthesis under each linkage group. Markers in blue denote common markers across different maps. Two anthocyanin candidate MYB genes, *DcMYB6* and *DcMYB7*, are denoted in bold purple letters.

In a previous study by Cavagnaro et al. (2014), we reported on a framework QTL map of 70349 (*N* = 187) with co-localized QTL for root anthocyanins in two regions of chromosome 3 (Figure 2A). One of these regions harbored 5 major QTL which explained most of the variation, including QTL for root total pigment estimate (named *RTPE-Q1*) and four individual root anthocyanins, as determined with HPLC analysis. In the present study, phenotypic data for RTPE and root anthocyanin pigments from 234 additional 70349 plants, for a total of 421 F_2_ plants, were used along with genotypic data from 15 SNP markers in the *RTPE-Q1* region, to construct a linkage map with better resolution of this QTL. The resulting 18.4 cM linkage map harbored co-localized QTL for *RTPE-Q1* and four anthocyanin glycosides (Cy3XG, Cy3XGG, Cy3XSGG, and Cy3XFGG) (Figure 2B). All five QTL had strong statistical supports (LOD = 29.2–67.9) and large effects on phenotype, explaining 37.1% (Cy3XGG) to 52.3% (*RTPE-Q1*) of the observed variation (Table 2).

**Table 2:** Summary of QTL for root total pigment estimate (RTPE) and anthocyanin pigments (Cy3XG, Cy3XGG, Cy3XSGG, and Cy3XFGG) fine-mapped in the RTPE region of chromosome 3 in population 70349.

Trait	QTL ID^∗^	Position (cM)	LOD value	1.5 LOD support interval	Nearest marker	% variation explained
RTPE	RTPE-Q1	10.1	50.6	8.7–11.3	K0653	52.3
Cy3XG	Cy3XG-Q1	10.3	29.3	8.3–11.3	K0627, K2590	38.6
Cy3XGG	Cy3XGG-Q2	7.1	29.2	5.0–9.0	K2309	37.1
Cy3XSGG	Cy3XSGG-Q1	10.3	48.9	8.7–11.3	K0627, K2590	44.5
Cy3XFGG	Cy3XFGG-Q1	10.3	67.9	8.7–11.3	K0627, K2590	51.2

*^∗^The same IDs were used as in the framework QTL map reported by Cavagnaro et al. (2014). Cy3XG: Cy-3-(2″-xylose-galactoside), Cy3XGG: Cy-3-(2″-xylose-6-glucose-galactoside), Cy3XSGG: Cy-3-(2″-xylose-6″-sinapoyl-glucose-galactoside), Cy3XFGG: Cy-3-(2″-xylose-6″-feruloyl-glucose-galactoside*.

The map region delimited by the QTL confidence intervals was substantially smaller in the new map, constructed using a larger population size (*N* = 421), as compared to the original map (*N* = 187). Together, these 5 QTL spanned a 12 cM region in the framework map (Cavagnaro et al., 2014 and Figure 2A), whereas in the new map they spanned a 6.3 cM region, with co-localized QTL for RTPE and three root anthocyanins (Cy3XG, Cy3XSGG, and Cy3XFGG) within a 3 cM region (Figure 2B and Table 1). Interestingly, *DcMYB6*, an R2R3-MYB gene recently associated with anthocyanin accumulation in the carrot root (Xu et al., 2017), mapped within the *RTPE-Q1* QTL support interval (Figure 2B).

**FIGURE 3:**
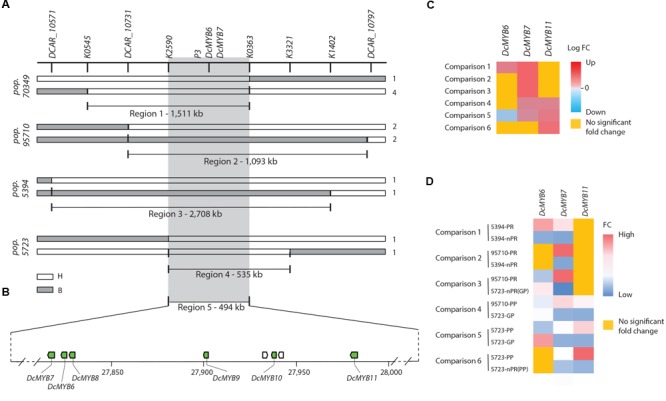
Fine mapping summary and comparative transcriptome analysis. **(A)** Haplotypes delimiting the genomic regions controlling the *RTPE-Q1* QTLs in 70349 (region 1) and the *P_3_* locus in 95710, 5394 and 5723 (regions 2–4). Genotyping scores to draw this figure were extracted from Supplementary Figure S2. The white bars indicate the heterozygous haplotypes (H = *Aa*) and the gray bars indicate the homozygous recessive haplotypes (B = *aa*). Region 5 represent the genomic sequence delimited by the nearest markers flanking the *RTPE-Q1* QTL and the *P_3_* locus across regions 1–4. Numbers on the right side of each bar represent the number of recombinant genotypes for each haplotype. **(B)** Schematic representation of carrot chromosome 3 containing regions 1–5 and the six anthocyanin related *DcMYB6-11* (green boxes). The scheme was drawn to scale. **(C)** RNASeq results for *DcMYB6-7-11* across 6 comparisons: The heatmap represents the log fold changes (Log FC) of expression level for each comparison. Positive values (>0, from light red to dark red) indicate down-regulation, negative value (<0, from light blue to dark blue) indicates up-regulation. Genes not differentially expressed across the 6 comparisons are highlighted in yellow. **(D)** qRT-PCR results for *DcMYB6-7-11* across 6 comparisons. The heatmap represent the average mean fold changes in gene expression normalized to internal control genes ACTIN. The value in each cell was calculated by averaging the qRT-PCR results obtained from 3 biological replication and three technical replications. qRT-PCR normalized by EF-1α were consistent with the results presented here and are included in Supplementary Figure S6. Genes not differentially expressed are highlighted in yellow.

Analysis of the genotypic scores for the SNP markers in the map region containing *RTPE-Q1*, delimited by *RTPE-Q1*-flanking markers K0702 and K3153 (Figure 2B), revealed 8 recessive (*aa*) to dominant (*A_*) recombinant genotypes. Alignment of the markers in the *RTPE-Q1* region (as defined above) against the carrot genome assembly was used initially to delimit the corresponding genome sequence and to develop new markers, within this region, for further resolution of the position of *RTPE-Q1*. Thus, three additional markers, two SNPs (K0545, K0363) and one indel (Pindel2), were developed and used to identify the recombination breakpoints in these eight genotypes, enabling us to narrow down the genomic region harboring *RTPE-Q1* to a 1,511 Kb region (Figures 3A,B and Supplementary Figure S2). This region, as delimited by markers K0363 and K0545 (Figures 2B, 3A), was named “region 1” (Figure 3). Region 1, which contained 116 predicted genes, was used to search for candidate genes for *RTPE-Q1*. The recently described MYB gene *DcMYB6* (Xu et al., 2017), which was not anchored to carrot pseudomolecules in the carrot genome assembly (Iorizzo et al., 2016), would be expected to be physically located within this region.

### Fine Mapping and Delimitation of the Genomic Region Harboring *P*_3_ in Diverse Genetic Backgrounds

A total of 22 SNP markers developed from the region of the carrot genome sequence corresponding to the *RTPE-Q1* confidence interval, were mapped in three populations segregating for *P*_3_, the locus conditioning root and petiole anthocyanin pigmentation. High resolution linkage maps were obtained for the three mapping populations (Figures 2C–E). Analysis of markers order revealed high collinearity across the three maps, as can be observed in Figures 2C–E, and as indicated by the Spearman rank correlation value of 1.00 obtained for markers order among maps.

*P*_3_ was tightly linked (≤3.2 cM) to all SNP markers mapped. The SNP marker for *DcMYB6* was polymorphic in 95710 and completely co-segregated with *P*_3_ (Figure 2C). In the 5394 and 5723 maps, the closest marker was K0363, which mapped at 0.1 cM from *P*_3_ in 5394 and at 0.2 cM from *P*_3_ in 5723, and was also tightly linked to *P*_3_ in 95710, at a distance of 0.1 cM from the trait locus. The smallest map interval containing *P*_3_, as defined by the closest flanking markers of the trait locus (not considering the fully co-segregating marker *DcMYB6*), spanned 0.8 cM in 95710, 0.3 cM in 5364, and 0.8 cM in 5723. Considering the estimated size of the carrot genome (473 Mb) and length of the integrated linkage map used for assembling the carrot genome sequence (622 cM) (Iorizzo et al., 2016), the average sequence length per map unit is 0.76 Mb/cM. Thus, based on this estimate, the closest marker (K0363) to *P*_3_ was ∼76–152 kb from the trait locus, and the physical position of *P*_3_ in the carrot genome was within a 228–608 kb well-delimited region. It must be noted that, because *DcMYB6* fully co-segregated with *P*_3_ in 95710, we concluded that its physical location is within this genomic region.

In order to further resolve the genomic region containing *P*_3_, analysis of the genotypic scores for the SNP markers flanking *P*_3_ was performed to identify recessive (*aa*) to dominant (*A_*) recombination breakpoints associated with changes in the purple color phenotype of carrot roots and petioles in each of these populations (Figure 3A and Supplementary Figure S2). As a result, linkage blocks associated with the purple color phenotype were identified and recombination breakpoints were found on each side of *P*_3_, further delimiting the genomic region of *P*_3_ to 1,093 Kb in 95710 (from here on referred to as “region 2”), 2,708 Kb in 5394 (“region 3”), and 535 Kb in 5723 (“region 4”) (Figure 3A and Supplementary Figure S2). Sixty-eight predicted genes were found in region 2, 201 genes in region 3, and 29 genes in region 4. Genomic regions 1–4, harboring *RTPE-Q1* and *P*_3_, overlapped between markers K2590 and K0363, delimiting a 494 Kb region (referred to as “region 5”) (Figure 3A). These five genomic regions, as defined above, were further analyzed in detail for the identification of candidate genes controlling root and petiole pigmentation in the *RTPE-Q1* and *P*_3_ genomic region.

### Identification and Analysis of Candidate Genes for Root and Petiole Phenotypes in the *RTPE-Q1* and *P*_3_ Region

No structural genes involved in the flavonoid or anthocyanin biosynthetic pathways were found in genomic regions 1–5 associated with *RTPE-Q1* and *P*_3_, as revealed by analysis of the coordinates of all the genes involved in these pathways from the carrot genome annotation (Iorizzo et al., 2016). Similarly, none of the carrot transcription factors previously reported to be associated with anthocyanin biosynthesis co-localized with the *RPTE-Q1*/*P*_3_ region (Supplementary Table S7). Among those previously reported TFs, *DcMYB6* (Xu et al., 2017) was found in a small contig (C10735702, length = 3,207 bp) that had previously failed to be included –by the scaffolding pipeline used- in the carrot genome pseudomolecules (Iorizzo et al., 2016) and therefore its physical position could not be established directly, as done for the other TFs. Thus, in order to determine the physical position of *DcMYB6*, we aligned contig C10735702 sequence along with Illumina Paired-end (PE) sequences (Iorizzo et al., 2016) to the carrot genome assembly (Supplementary Figure S3). The first 1,218 bp on the left side of contig C10735702 uniquely mapped (100% similarity) to an anchored contig with coordinates 27,830,907–27,834,373 of chromosome 3, at its junction with a gap sequence (filled with Ns). Two-kb, 5, 10, 20, and 40 kb PE data unambiguously linked contig C10735702 to the left and right side of the 3,207-nt existing gap, demonstrating that this contig can be anchored at this position of carrot chromosome 3. Therefore, the sequence of contig C10735702 filled the existing gap at this junction, and it was assigned the following coordinates in chromosome 3: DCARv2_Chr3:27,830,908–27,834,114 (Supplementary Figure S3). In conclusion, *DcMYB6*, which corresponds to gene DCAR_000385 and was originally predicted within contig C10735702, is located at position 27,831,723–27,833,545 of chromosome 3. This region overlaps with regions 1–5, which were determined by fine mapping of the *RTPE-Q1* and *P*_3_ region (Figures 3A,B).

In addition to defining the genetic and physical position of *DcMYB6* in the region of *RTPE-Q1* and *P*_3_, and thereby considering this TF as a putative candidate gene, we performed a comprehensive analysis of the genes in these five regions, including gene prediction, orthologous and phylogenetic analyses, to identify other anthocyanin-related TFs.

Genome wide prediction of carrot TFs belonging to four gene families, namely MYB-HB-like, WD40-like, MYB/SANT and bHLH, identified 891 TFs, with a predominance of WD40-like (346 genes) and MYB-HB-like genes (325 genes) (Supplementary Table S10). Fourteen carrot TFs (7 MYB-HB-like and 7 bHLH) were found in one or more of the genomic regions associated with *RTPE-Q1* and *P*_3_, and 8 of them (6 MYB-HB-like and 2 bHLH) were present in all five of these regions (Supplementary Table S11).

Orthologous analysis using these 14 carrot TFs and all of the TFs predicted for the four TF families from other plant taxa revealed eleven carrot genes clustered into seven orthologous groups (OG1-OG7), whereas the remaining 3 TFs remained unclustered (singletons) (Supplementary Table S12). The seven orthologous groups comprised 332 genes from 54 organisms with extremely variable degree of phylogenetic relatedness to carrot, ranging from vegetable crops within the same Asterids clade (e.g., tomato and pepper) to marine phytoplankton and unicellular algae (Supplementary Table S13). Four carrot TFs grouped in OG1 (including *DcMYB6*), one TF in each of OG2-OG6, and two TFs in OG7. A search for previously reported functionally characterized genes within each OG revealed that OG1 and OG5 comprised R2R3-MYB transcription factors involved in transcriptional regulation of anthocyanin biosynthesis in model (*Arabidopsis thaliana*) and crop species (grapevine, tomato, apple, and eucalyptus), whereas OG2, OG3, OG4, and OG6 clustered TFs with biological functions unrelated to anthocyanin biosynthesis, such as meristem formation (OG2 and OG3), development of root hairs (OG4) and development of stomatal complex (OG6) (Supplementary Table S14). OG7 was composed of two TFs from carrot with uncharacterized function. Altogether, these results suggest that 5 of the 14 TFs found in the *RTPE-Q1* and *P*_3_ genomic region, namely those included in OG1 (DCAR_010745, DCAR_010746, DCAR_010747, and DCAR_000385) and OG5 (DCAR_010751), are potentially involved in the regulation of carrot anthocyanin biosynthesis. These five TFs are MYB genes and correspond to the R2R3-MYB gene family. Our results also suggest that four carrot TFs (TFs included in OG2, OG3, OG4, and OG6) have functions unrelated to anthocyanin biosynthesis. The other 5 carrot TFs (three singletons and two TFs in OG7) could not be associated with any anthocyanin-related TF from other species based on their transcript sequence similarities and, therefore, one cannot speculate on their putative function.

Phylogenetic analysis including the 7 carrot MYB-HB-like (R2R3-MYB) genes identified in the *RTPE-Q1* and *P*_3_ genomic regions (Supplementary Tables S11, S12) and 41 R2R3-MYBs from other plant species involved in the regulation of anthocyanin, proanthocyanidin, and flavonoid biosynthesis (Supplementary Table S5) revealed that six carrot MYBs (namely, DCAR-010745, DCAR_010746, DCAR_010747, DCAR_010749, DCAR_010751, and DCAR_000385) were included in a clade of MYBs involved in the regulation of anthocyanin biosynthesis (Supplementary Figure S4). This clade of anthocyanin-related R2R3-MYBs included the following genes: tobacco (*Nicotiana tabacum*) *NtAN2*; petunia (*Petunia hybrida*) *PhAn2*; tomato (*Solanum lycopersicum*) *LeANT1*; sweet potato (*Ipomoea batatas*) *IbMYB1*; morning glory (*Ipomoea nil*) *InMYB2*; grapevine (*Vitis vinifera*) *VvMYBA1* and *VvMYBA*2; blood orange (*Citrus sinensis*) *CsRuby*; snapdragon (*Antirrhinum majus*) *AmVENOSA, AmROSEA1*, and *AmROSEA2*; *A. thaliana AtPAP1, AtPAP*2, and *AtMYB114*; mangosteen (*Garcinia mangostana*) *GmMYB10*; Chinese bayberry (*Myrica rubra*) *MrMYB*1; apple (*Malus? × ?domestica*) *MdMYB10a* and *MdMYB1-1*; *Gerbera hybrida GhMYB10*; *Medicago truncatula MtLAP1*; *Lilium hybrid LhMYB6*; and *Epimedium sagittatum EsMYBA1*. These results largely coincide with those from the orthologous analysis, considering that five of the six carrot MYBs phylogenetically associated with anthocyanin-related MYBs correspond to the 5 carrot MYBs clustered in OG1 and OG5, the two OGs with TFs involved in anthocyanin biosynthesis.

A similar phylogenetic analysis as described above for MYB genes was performed using the 7 carrot bHLH TFs identified in the *RTPE-Q1*/*P*_3_ genomic regions (Supplementary Tables S11, S12) and 24 bHLH genes from other species, revealing that none of the carrot bHLH TFs grouped with anthocyanin-related bHLH from other species (Supplementary Figure S5). These results suggest that the carrot bHLH genes positionally associated with *RTPE-Q1* and *P*_3_ are not involved in anthocyanin biosynthesis.

The results from the orthologous and phylogenetic analyses identified six R2R3-MYB TFs potentially involved in the transcriptional regulation of carrot anthocyanins biosynthesis. The fact that all of the carrot MYBs were clustered with transcriptional activator MYBs in both analyses, and that they were unrelated to MYBs with transcriptional repression activity, suggest that they may also function as activators of the anthocyanin pathway in carrot.

These six carrot *MYB* genes are located within a 166 kb region, with four of them being organized in tandem within a region spanning 86 kb (Figure 3B). These genes were denominated as follows: *DcMYB6* (DCAR_000385), *DcMYB7* (DCAR-010745), *DcMYB8* (DCAR_010746), *DcMYB9* (DCAR_010747), *DcMYB10* (DCAR_010749), and *DcMYB11* (DCAR_010751). Finally, linkage mapping of DCAR_010745 (from here on named *DcMYB7*) and *DcMYB6* in 70349 and 2170, the mapping populations originally used to map the *RTPE-Q1* and *P*_3_ loci, respectively (Cavagnaro et al., 2014), confirmed co-localization of these two genes with *RTPE-Q1* and *P*_3_ (Figures 2B,F). Considering these results, these six MYB TFs are considered candidate genes for root and petiole pigmentation in the *RTPE-Q1* and *P*_3_ genomic region (Supplementary Table S15).

### Transcriptome Analysis (RNA-Seq)

Genome wide quantitative transcriptome analysis was performed in selected samples representing purple pigmented and non-pigmented root and petiole tissues from progeny of the mapping populations segregating for *P3* used in this study (populations 5394, 5723, 95710). After cleaning low quality reads, 43–55 million high-quality reads per biological replicate were retained (Supplementary Table S8) for further downstream analysis. In total, 6 pairwise comparisons of purple versus non-purple root and leaf tissues were performed to identify differentially expressed genes (DEGs) from the five genomic regions harboring *RTPE-Q1* and *P*_3_ (Supplementary Tables S16–S19). *DcMYB8* and *DcMYB9* were not differentially expressed in any of the six comparisons. Given that the 5394 F_3_ population derived from a self-pollinated plant of 70349, comparisons of 5394 purple versus non-purple roots (comparison 1), were used to identify DEGs in the 1,511 and 2,708 kb fine mapped region 1 (70349) and region 3 (5394), respectively. In total, 8 and 12 DEGs were identified in region 1 and region 3, respectively. Two anthocyanin-related MYBs, *DcMYB6* and *DcMYB7*, were upregulated in all of the purple root samples analyzed (Figure 3C and Supplementary Tables S16, S18). To identify DEGs in the 1,093 kb ‘region 2’ of population 95710, plants with the purple root phenotype were compared with non-purple roots of 5394 (comparison 2) and with non-purple (orange) roots of 5723 (comparison 3). In addition, purple petioles from plants of 95710 were compared with green petioles of 5723 (comparison 4). In total, 15, 14, and 12 DEGs were identified in the 95710 comparisons 2, 3, and 4, respectively (Supplementary Table S17). *DcMYB7* was upregulated in the purple tissues in all of the comparisons performed, while *DcMYB11* was only upregulated in comparison 4 (Figure 3C and Supplementary Table S17).

Transcriptome analysis comparing purple petioles *versus* green petioles of plants from 5723 (comparison 5) were used to identify DEGs within the 535 kb fine mapped ‘region 4’. In total, 8 DEGs were identified in comparison 5, with 5 genes being upregulated and 3 genes downregulated in purple petioles. These 8 DEGs identified in comparison 5 included *DcMYB6*, which was downregulated in purple petioles, and *DcMYB7, DcMYB10*, and *DcMYB11*, all upregulated in purple petioles (Figure 3C and Supplementary Table S19).

Altogether, results from the transcriptome analysis revealed that four of the six anthocyanin related MYBs identified in genomic regions 1 to 4 associated with *RTPE-Q1* and *P*_3_ were differentially expressed in at least one comparison (Figure 3B). *DcMYB7* was the only gene upregulated in all of the anthocyanin-pigmented tissues of both roots and petioles in all the comparisons performed. *DcMYB11* was specifically and consistently upregulated in purple petioles (comparisons 4 and 5). *DcMYB6* was upregulated in purple roots of 5394 (comparison 1) but was also upregulated in the non-purple roots with green petioles of 5723 (comparisons 5). *DcMYB10* was upregulated only in purple petiole samples of 5723 (comparison 5). Comparison between purple petiole versus non-purple (orange) root of the same plants from population 5723 (comparison 6) further confirmed that *DcMYB11* was upregulated in purple petioles, whereas the transcript levels of this gene were nearly undetectable in the non-purple root of the same plants (Figure 3B and Supplementary Table S19).

### Flavonoid and Anthocyanin Gene Analysis

Given the multiple purple and non-purple tissue comparisons used in this study, transcriptome data were used to gain preliminary insights on the expression of the annotated structural genes from the flavonoid and anthocyanin biosynthetic pathways (Iorizzo et al., 2016) in the carrot root and petioles. For qualitative analysis (i.e., whether a gene is expressed or not), a gene was designated as ‘expressed’ if its RPKM (reads per kilobase of transcript per million mapped reads) value in any sample was greater than 1 (Supplementary Table S20). Overall, 11 structural genes (*PAL1, PAL3, PAL4, 4CL3-1, 4CL3-2, ACC1-1, C4H-1*, and 4 UFGT-like genes) were expressed in all the samples analyzed, whereas 8 genes (7 UFGT-like and 1 C4H-2) were not expressed in any of the samples. None of the structural genes were consistently differentially expressed in all the purple versus non-purple root comparisons. Four UDPG-like genes were specifically expressed in the petioles, but their expression was not associated with a particular petiole color phenotype.

Considering DEGs (quantitative analysis), the pattern of over-expressed genes in purple petioles and purple root was very different. Three genes (*CHS-1, DFR-1*, and *PAL-3*) were consistently upregulated in purple roots, in all the purple vs. non-purple root comparisons (comparisons 1–3), and one gene (*UDPG-like 4*) was consistently upregulated in purple petiole samples relative to non-purple petiole ones (comparisons 4 and 5) (Supplementary Table S20).

### Validation of Candidate Genes for Root and Petiole Pigmentation in the *RTPE-Q1* and *P*_3_ Genomic Region by RT-qPCR Analysis

To validate the RNA-Seq-based gene expression profiles, the expression levels of *DcMYB6, DcMYB7*, and *DcMYB11* were examined using RT-qPCR analysis. In all the ‘purple vs. non-purple root’ comparisons, *DcMYB7* was the only gene that consistently showed a significantly higher level of expression (Figure 3D and Supplementary Figure S6A). In all the ‘purple petiole vs. green petiole’ comparisons, *DcMYB7* and *DcMYB11* both had significantly higher expression levels (Figure 3D and Supplementary Figure S6B). In ‘purple petiole vs. purple root’ comparisons, *DcMYB11* had significantly higher expression levels in the petioles, whereas transcript levels were almost undetectable in the root (Figure 3D). Overall, RT-qPCR validated the RNA-Seq results and strengthened the candidacy of *DcMYB7* and *DcMYB11* for the genetic control of *P*_3_ and *RTPE-Q1*. *DcMYB7* is a candidate gene for anthocyanin pigmentation in the root of all the purple carrot sources used as progenitors in the mapping populations used in this study (see Table 1 for purple root sources and their geographical origins). *DcMYB11* is the best candidate gene for the genetic control of anthocyanin pigmentation in the petioles. In addition, given that *DcMYB7* is co-expressed (at low levels) with *DcMYB11* in purple petioles, the latter gene may act also as a co-regulator of anthocyanin pigmentation in the petioles. Finally, *DcMYB6*, a gene recently proposed to regulate anthocyanin biosynthesis in the carrot root (Xu et al., 2017), may regulate or co-regulate anthocyanin pigmentation in specific genetic backgrounds (e.g., 5394), but not in others.

## Discussion

### The *P*_3_ Region Controls Different Tissue-Specific Patterns of Anthocyanin Pigmentation in Carrot Roots and Petioles, Depending on the Genetic Background

To date, three simply inherited dominant loci controlling anthocyanin pigmentation in different parts of the carrot plant have been described. These correspond to *P*_1_, controlling pigmentation in the tap roots of B7262, a carrot line with purple color originating from eastern Turkey (Simon, 1996; Vivek and Simon, 1999; Yildiz et al., 2013); *P*_2_, conditioning pigmentation in the nodes in two genetic backgrounds from Turkey (Simon, 1996); and *P*_3_, which controls pigmentation in the root and petioles in the P9547 and PI652188 genetic backgrounds, purple carrot sources of Turkish and Chinese origins, respectively (Cavagnaro et al., 2014). In addition, QTL for total root pigmentation (*RTPE-Q1*) and four individual anthocyanin pigments were mapped and co-localized with *P*_3_ in the P9547 background (Cavagnaro et al., 2014). In the present study, based on high-resolution comparative mapping analysis, we demonstrated that *P*_3_ also conditions anthocyanin pigmentation in the root and petioles of BP85682, a purple carrot source from Syria. Thus, with the exception of B7262, *P*_3_ conditions anthocyanin pigmentation in the root and petioles in all the purple carrot genetic backgrounds examined to date. High-resolution comparative mapping analysis also confirmed that the *RTPE-Q1* and *P*_3_ loci correspond to the same map region, as indicated by the complete linkage found for two common markers, *DcMYB6* and *DcMYB7*, to both *RTPE-Q1* (in population 70349, Figure 2B) and *P*_3_ (in populations 95710 and 2170; Figures 2C,F), and by the tight linkage (≤0.2 cM) of marker K0363 to *RTPE-Q1* (in population 70349, Figure 2B) and *P*_3_ (in populations 95710, 5394, and 5723; Figures 2C–E).

In addition, fine mapping of QTL in population 70349 confirmed previous results of Cavagnaro et al. (2014) reporting co-localization of *RTPE-Q1* and four major QTL for individual anthocyanin pigments, and further narrowed this map region from 12 to 6.3 cM, with four of the QTL (including *RTPE-Q1*) being within a 3 cM region. This increase in map resolution in the *RTPE-Q1* region, as consequence of using a larger population size and more markers, has also proven successful for other QTL and species (Chen et al., 2016; Yeo et al., 2017), and facilitated the search of candidate genes for anthocyanin pigmentation to a more-confined genomic region.

### The Expression Pattern of a Set of Fine Mapped R2R3-MYB Genes Is Associated With Anthocyanin Accumulation in the Carrot Root and Petiole

As summarized above, previous studies on anthocyanin genetics in carrot mainly focused on inheritance of the trait(s) and linkage mapping of qualitative (*P*_1_, *P*_3_) and quantitative (QTL for RTPE and for individual anthocyanins pigments of the root) traits. In addition, a few studies used a candidate gene approach to investigate anthocyanin regulation in carrot. Yildiz et al. (2013) mapped six structural anthocyanin biosynthetic genes [phenylalanine ammonia-lyase (*PAL3*), chalcone synthase (*CHS1*), flavanone 3-hydroxylase (*F3H*), dihydroflavonol 4-reductase (*DFR1*), leucoanthocyanidin dioxygenase (*LDOX2*), and UDP-glucose:flavonoid 3-O-glucosyltransferase (*UFGT*)] and 3 regulatory genes (*DcEFR1, DcMYB3*, and *DcMYB5*) in a population segregating for *P*_1_, which conditions purple root color in B7262, but none of these genes cosegregated with the trait locus, suggesting that they are not candidates for *P*_1_. Ozeki et al. (2000), Maeda et al. (2005), and Wako et al. (2010), identified five MYB transcription factors, named *DcMYB1* to *DcMYB5*, and used carrot cell suspension cultures to study their involvement in the transcriptional regulation of phenylalanine ammonia–lyase genes (*DcPAL1* to *DcPAL4*). Together, their results indicated that *DcMYB1, DcMYB3*, and *DcMYB5* were the strongest transcriptional activators of PAL genes in carrot cell cultures, suggesting that they may play important roles in carrot anthocyanin biosynthesis. However, these studies were performed *in vitro* using carrot protoplasts, and the possibility remains that these results may not be extrapolable to *in planta* conditions, such as the carrot root.

More recently, Xu et al. (2017) studied the expression of a new anthocyanin related MYB, *DcMYB6*, in purple and non-purple carrot roots, and transformed *A. thaliana* with this gene to characterize its function. The expression pattern of *DcMYB6* was correlated with anthocyanin production in the carrot root, and its overexpression in *Arabidopsis* led to enhanced anthocyanin accumulation in both vegetative and reproductive tissues. However, because their studies did not include linkage or physical localization data for this gene relative to the trait locus (e.g., *P*_1_, *P*_3_, or another/new root pigmentation locus), the possibility remains that other regulatory or structural anthocyanin genes may be involved –along with *DcMYB6*- in the regulation of root anthocyanin pigmentation. It must also be noted that the purple carrot genetic background used by Xu et al. (2017) does not correspond -based on the cultivar name and phenotypic descriptions reported by them- to any of the materials used in the present study.

Here we report the first study in carrot that integrates association mapping and candidate gene identification for anthocyanin accumulation in root and petioles across three different genetic backgrounds. We delimited the region of *P*_3_ and *RTPE-Q1* to a 494 Kb-region of the long arm of chromosome 3. A comprehensive analysis that integrated prediction of transcription factors, orthologous and phylogenetic analysis identified a cluster of six anthocyanin related R2R3-MYB genes in this region, which included *DcMYB6* but none of the other MYB genes (*DcMYB1* to *DcMYB5*) previously reported in carrot (Ozeki et al., 2000; Maeda et al., 2005; Wako et al., 2010). Comparative transcriptome (RNAseq) and gene expression (qRT-PCR) analyses strongly suggest that two of these newly identified R2R3-MYBs, namely *DcMYB7* and *DcMYB11*, control anthocyanin pigmentation in carrot root and leave petioles. *DcMYB7* was the only gene upregulated in all purple tissues from root and petioles, while *DcMYB11* was exclusively upregulated in all the purple petiole tissues. Given these results we hypothesize that *DcMYB7* is a key gene controlling anthocyanin pigmentation in the carrot root, whereas *DcMYB11* specifically regulates or co-regulates (with *DcMYB7*) petiole pigmentation.

The gene expression pattern of *DcMYB6* across the different comparisons performed was, altogether, not positively correlated with anthocyanin pigmentation in neither root nor petioles. While this gene was upregulated in purple roots of population 5394, it was downregulated in purple roots of population 95710 and in purple petioles of population 5732 and 95710 (Supplementary Figures S6A,B). These data indicate that *DcMYB6* is not the key gene controlling anthocyanin pigmentation in either tissue type in the carrots used in this study. In addition, these data suggest a genotype-dependent and/or tissue-specific activity for this gene. In line with this hypothesis, it should be noted that the purple carrot cultivars used by Xu et al. (2017) accumulated anthocyanins across the entire root section (i.e., in both root tissues, phloem and xylem), whereas all the mapping populations used in the present study, except for 5394 (a derivative F_3_ from 70349), had purple roots with anthocyanins in the outer-most cell layers of the phloem, but not in the inner-phloem or xylem tissues (see Supplementary Figure S1). On the other hand, purple carrots of population 5394, with anthocyanin pigmentation in both the phloem and xylem root tissues, revealed overexpression of *DcMYB6* (as compared to non-purple roots of the same population), coincidently with the results of Xu et al. (2017). Thus, it is possible that *DcMYB6* is involved in tissue-specific regulation of anthocyanin biosynthesis in the inner root tissues, with *DcMYB7* controlling anthocyanin pigmentation in the root outer-phloem and in petioles. The activity of *DcMYB6* in the petioles of the carrot cultivars used by Xu et al. (2017) was not assessed in their work and therefore direct comparisons cannot be made with our results.

The regulatory mechanisms underlying anthocyanin accumulation in plants usually involves direct interactions between transcription factors and structural genes involved in flavonoid/anthocyanin biosynthesis (Liu et al., 2018). In carrot, 97 structural genes of the flavonoid/anthocyanin biosynthetic pathway have been annotated (Iorizzo et al., 2016). Although two studies have investigated their expression levels in purple versus non-purple carrot roots (Yildiz et al., 2013; Xu et al., 2014) a direct association of their expression patterns with specific regulatory genes has not been established. In the current study, seven genes annotated as UFGT-like and one C4H-2 gene were not expressed in any of the carrot samples analyzed, suggesting that these genes are either not involved in the anthocyanin biosynthetic pathway in carrot root and petioles, or they represent pseudogenes. Overall, none of the structural genes examined was specifically expressed in purple tissues of the root or petiole, indicating that the purple phenotype is likely a result of overexpression in genes of the flavonoid or anthocyanin biosynthetic pathway rather than an on/off switch mechanism. This observation further supports the candidacy of one or more transcription factors which, by means of transcriptional regulation of structural genes, modulate anthocyanin accumulation in carrot root and petioles.

Three genes, *CHS-1, DFR-1*, and *PAL-3*, were over-expressed in all purple root samples, while a UFGT-like gene was upregulated in all purple petiole samples. The different patterns of DEGs identified in purple petiole and purple root samples suggest that different molecular mechanisms control these two phenotypes. In a previous study, five structural genes (*CHS-1, DFR-1, F3H-1, LDOX-2*, and *PAL-3*) were found to be upregulated in solid purple carrots as compared to non-purple carrots (Yildiz et al., 2013). Similar results were obtained by Xu et al. (2014), reporting upregulation of nine structural genes (*PAL3/PAL4, C4AH1, 4CL1, CHS1, CHI1, F3H1, F3’H1, DFR1*, and *LDOX1/LDOX2*) in purple-rooted carrots as compared to non-purple ones. Recently, Xu et al. (2017) found that *DcMYB6* induce upregulation of three of these *Arabidopsis* homologous, *AtDFR, AtCHS*, and *AtUGT78D2*. Interestingly, these three genes (CHS, DFR, and UFGT) have been demonstrated to be a direct TF targets to regulate anthocyanin biosynthesis in other plants (Liu et al., 2018) making them candidate targets for future studies aiming at elucidating the molecular mechanism underlying the regulation of anthocyanin accumulation in carrot.

This study links, for the first time, candidate regulatory genes with anthocyanin pigmentation in specific root and leaf tissues. In addition, the concomitant overexpression of *DcMYB7* and structural anthocyanin genes *CHS-1, DFR-1*, and *PAL-*3 in purple roots suggests a transcriptional regulatory role for *DcMYB7* upon the latter genes. Similarly, the coincident upregulation of *DcMYB7* and *DcMYB11* with a UFGT-like gene in purple petioles suggests that this UFGT is regulated by one or both of these MYBs. Further analysis of these genes in different plant tissues and genetic stocks will help elucidate their roles and interactions in regulating carrot anthocyanin pigmentation. In addition, ongoing research using carrot populations segregating independently for xylem and phloem anthocyanin pigmentation will likely reveal other genetic factors controlling root tissue-specific pigmentation.

### The Chromosomal Organization of the Carrot R2-R3-MYBs Into a Gene Cluster Is Common Among Plant Genomes

The six R2-R3-MYB genes described herein (*DcMYB7* to *DcMYB11*) are organized in cluster/tandem within a ∼166 Kb region of chromosome 3 (Figure 3B and Supplementary Table S15). Genome wide analyses of R2-R3-MYB family members have revealed that these transcription factors (and other MYB subfamilies) are commonly found in gene clusters in the genomes of many plant species, including *Arabidopsis* (Stracke et al., 2001), soybean (Du et al., 2012a), maize (Du et al., 2012b), poplar (Wilkins et al., 2009), grapevine (Matus et al., 2008), and cotton (Salih et al., 2016). The main causes of MYB gene-family expansion and their chromosomal localization in gene clusters have been attributed to tandem and segmental duplication events (Feller et al., 2011). Similarly, in carrot tandem and segmental duplications represented main mode of duplication of the MYB-HB-like TF that include R2-R3-MYB genes (Iorizzo et al., 2016), indicating that these same evolutionary forces have also shaped the genome organization and diversity of the R2-R3-MYB gene family in carrot.

## Data Availability Statement

The RNAseq datasets generated for this study can be found in the Genbank Short Read Archive acc. no. SRP156284.

## Author Contributions

MI designed the study. PS developed the plant populations. HB performed all bioinformatic analyses. YZ performed the genotyping analysis. JZ extracted the RNA and performed the Real time PCR. MI and PC interpreted the results. MI, PC, and HB drafted the sections of the manuscript and prepared the figures and tables. PS, YZ, and JZ critically revised the manuscript. MI and PC prepared the final version of the manuscript. All authors read, reviewed, and approved the manuscript.

## Conflict of Interest Statement

The authors declare that the research was conducted in the absence of any commercial or financial relationships that could be construed as a potential conflict of interest.

## Footnotes

1 https://www.lgcgroup.com/products/kasp-genotyping-chemistry/#.Wx5-8novxjU
