# Supplementary material for: A Cluster of MYB Transcription Factors Regulates Anthocyanin Biosynthesis in Carrot (Daucus carota L.) Root and Petiole
Source: Front Plant Sci. 2019 Jan 14;9:1927. doi: 10.3389/fpls.2018.01927 (PMC6339893; doi:10.3389/fpls.2018.01927)
Supplement: Supplementary file 2 [file Data_Sheet_1.PDF]

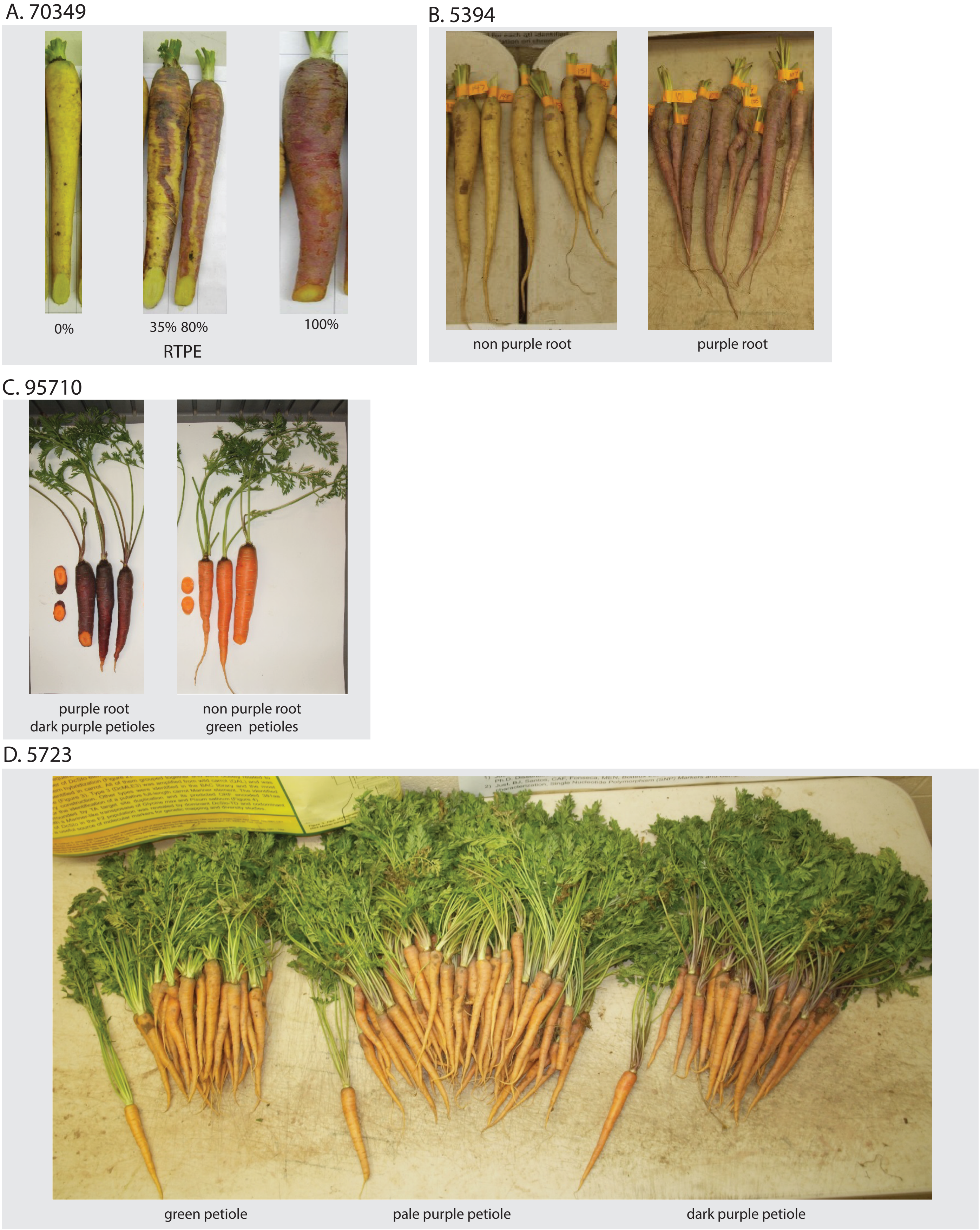

Supplementary Figure S1. Carrot root and leaf petiole color phenotypes in mapping populations 70349, 5394, 95710, and 5723. In population 70349, the percentage of purple pigmentation covering the root surface (called "RTPE" for "root total pigment estimate"), which estimates the root total pigment content in this genetic background, was scored visually as described by Cavagnaro et al. (2014) and varied from 0% to 100% (A). Root anthocyanin pigmentation in the other mapping populations was phenotyped based on the presence or absence of purple pigment (B-D). Petiole pigmentation was scored as the presence or absence of purple pigment in petioles of 5394 (B), 95710 (C) and 5723 (D). Additional phenotyping was performed in 5723 population, which segregated for green (GP), pale purple (pPP) and dark purple (dPP) petioles (D).

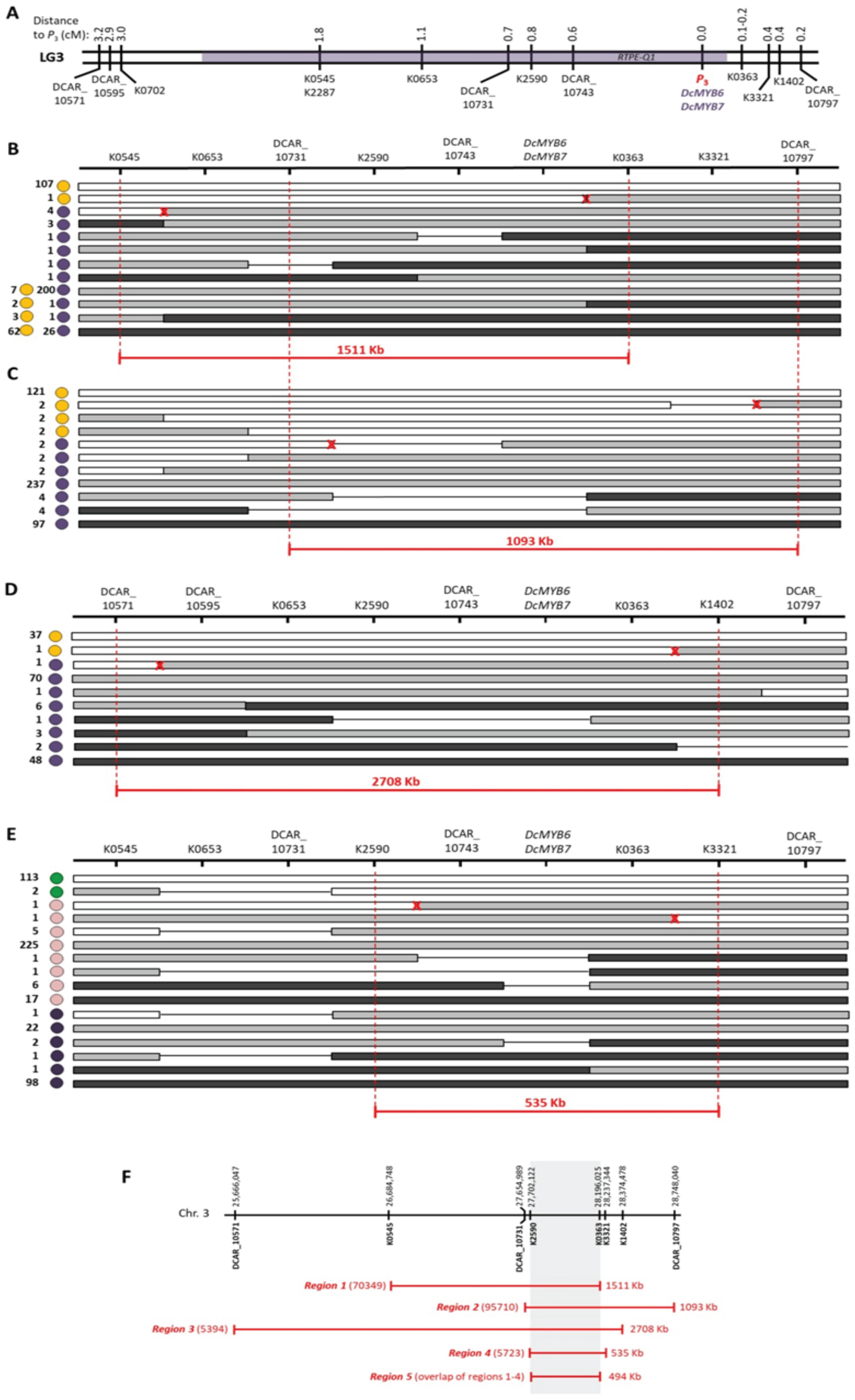

Supplementary Figure S2. Delimitation of the genomic region controlling anthocyanin pigmentation in carrot roots and petioles (P3). (A) Schematic representation of linkage group 3 (LG3) in the region of RTPE-Q1 and P3. Markers tightly linked to RTPE-Q1 and P3 from four linkage maps (see Figure 1 B-D) were included, and their genetic distance to P3 in their respective maps is indicated in centimorgans (cM). (B-E) Phenotypes, haplotype analysis and identification of linkage blocks associated with anthocyanin pigmentation in mapping populations 70349 (B), 95710 (C), 5394 (D), and 5723 (E). Homozygous recessive (aa), heterozygous (Aa), and homozygous dominant (AA) genotypes were indicated with white, gray, and black bars, respectively. Recessive (aa) to dominant (A_) recombination breakpoints closest to P3, and the genomic distance between their flanking markers (in kilobases), are indicated in red. Regions with no marker data are indicated with simple lines. Color phenotypes for root (B-D) and petioles (E) and the number of individuals associated with each genotype are indicated at the left of each bar. In 5394 (E), three phenotypic classes were considered for petiole color; green, pale purple and dark purple. (F) Summary of the resulting four genomic regions (Regions 1-4), and their overlap (Region 5), used for further analysis and identification of candidate genes for the genetic control of anthocyanin pigmentation in carrot roots and petioles.

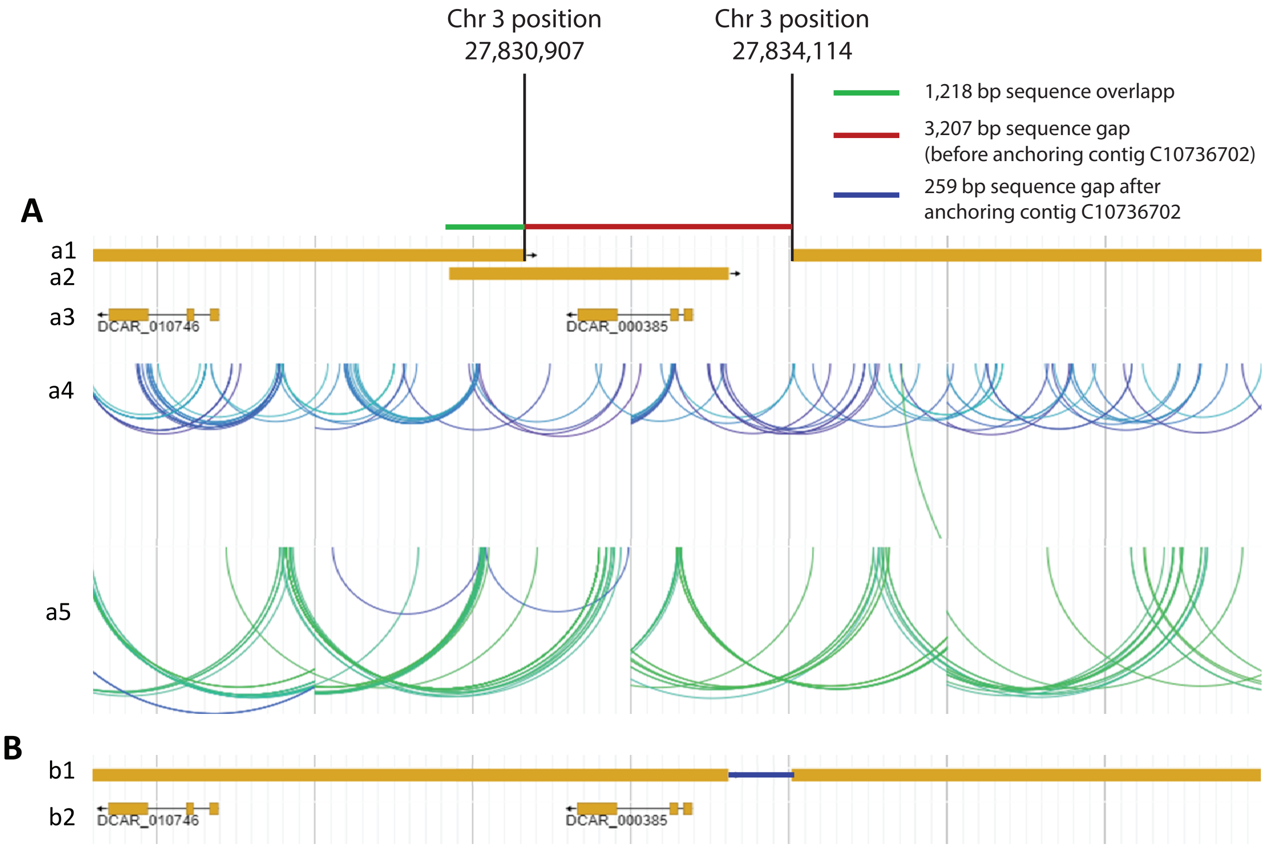

Supplementary Figure S3. Scheme of contig C10735702 anchoring strategy. Panel A: Jbrowse enlarged window of carrot chromosome 3 assembly v2.0 (Iorizzo et al., 2016), covering the mapping location of contig C10735702, before anchoring. a1-a2: indicates the mapping location of the un-anchored contig C10735702 on carrot chromosome 3. The red bar indicates a 3,207 bp gap existing at this location. The green bar indicates the 1,218 bp sequence of contig C10736702 that aligned within this region with 100% similarity. a3: indicates the predicted genes in this region. DcMYB6 corresponding to gene DCAR_000385 was predicted within contig C10735702. a4: 2 kb paired end reads (PE) that unambiguously aligned to this region. a5: 5Kb paired end reads (PE) that unambiguously aligned to this region. PE reads unambiguously link contig C10735702 to the left and right side of the 3,207 existing gap, demonstrating that this contig should be anchored at this location of carrot chromosome 3. Panel B: Jbrowse enlarged window of carrot chromosome 3 assembly v2.0 (Iorizzo et al., 2016), covering the mapping location of contig C10735702, after anchoring. b1: indicates the location of the carrot chromosome 3 including the sequence of contig C10735702. A gap of 259 bp on the right side was kept in the sequence (Supplementary file 1) to avoid any coordinate changes of the genomic sequence and genes upstream of the anchoring point. DcMYB6 is located at position 27,831,723-27,833,545 of chromosome 3.

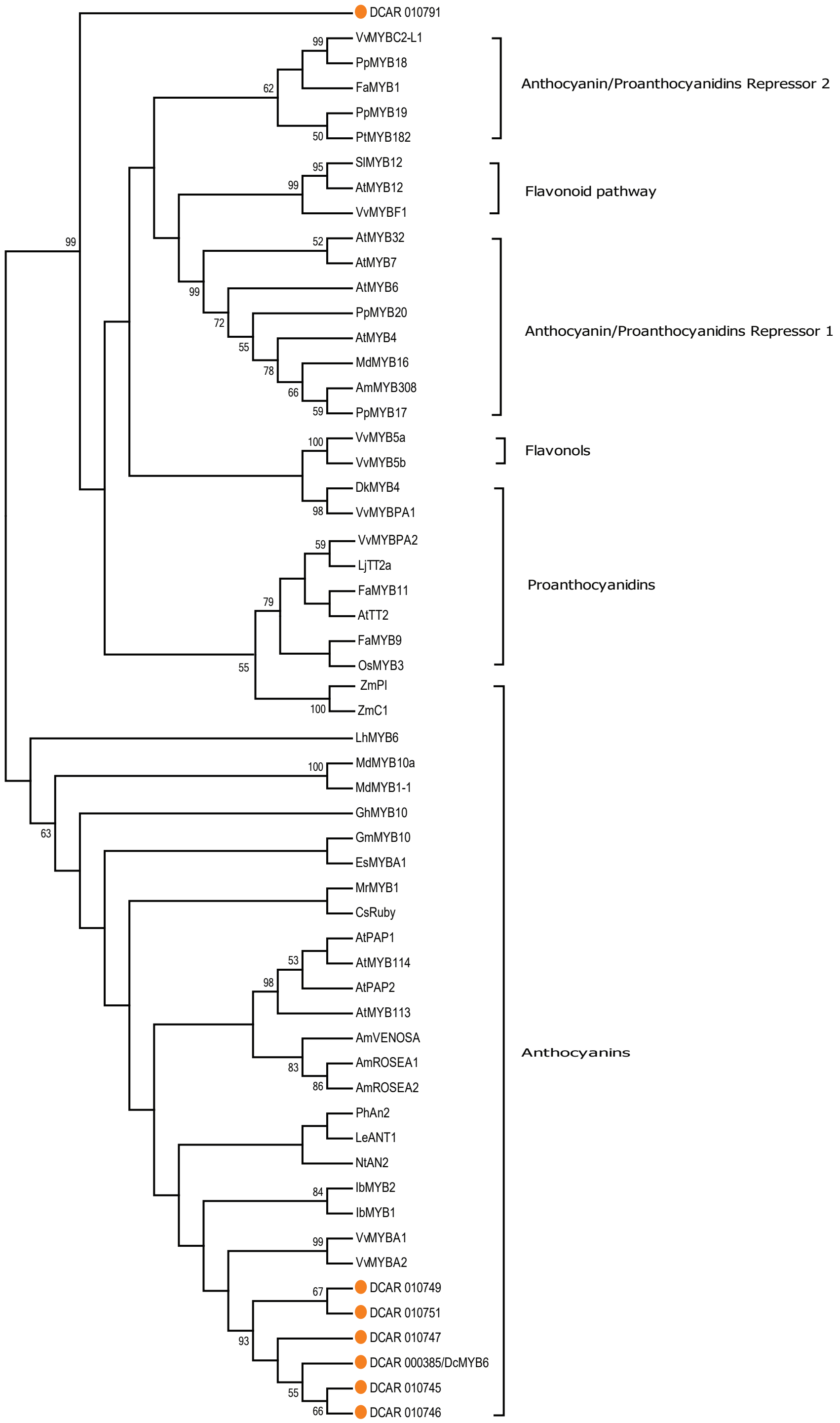

Supplementary Figure S4. Phylogenetic relationships among carrot R2R3-MYB genes located in the RTPE-Q1/P3 regions (MYB-HB-like) and R2R3-MYBs from other species involved in the regulation of different secondary metabolites. The tree was constructed using MEGA 7, neighbor-joining phylogeny testing, with 1,000 bootstrap replicates. The accession numbers and gene IDs of these proteins (or translated products) are as follows in the GenBank database: NtAN2, ACO52470; PhAn2, AAF66727; LeANT1, AAQ55181; IbMYB1, BAF45114; InMYB2, BAE94709; VvMYBA1, BAD18977; VvMYBA2, BAD18978; CsRuby, AFB73913; AmVENOSA, ABB83828; AmROSEA1, ABB83826; AmROSEA2, ABB83827; AtPAP2/MYB90, AAG42002; AtMYB114, Q9FNV8; AtPAP1/MYB75, AAG42001; AtMYB113, Q9FNV9; GmMYB10, ACM62751; MrMYB1, ADG21957; MdMYB10a, ABB84753; MdMYB1-1, DQ886414; GhMYB10, CAD87010; MtLAP1, ACN795410; LhMYB6, BAJ05399; EsMYBA1, AGT39060; ZmC1, AAA33482; ZmPl, AAA19819; LjTT2a, BAG12893; VvMYBPA2, ACK56131; FaMYB11, AFL02461; AtTT2, NP_198405; FaMYB9, AFL02460; OsMYB3, BAA23339; DkMYB4, BAI49721; VvMYBPA1, CAJ90831; AtMYB32, EFH43356; AtMYB4, NP_195574; AtMYB7, NP_179263; AtMYB12, ABB03913; SlMYB12, ACB46530; VvMYBF1, ACV81697; VvMYB5a, AAS68190; VvMYB5b, AAX51291.

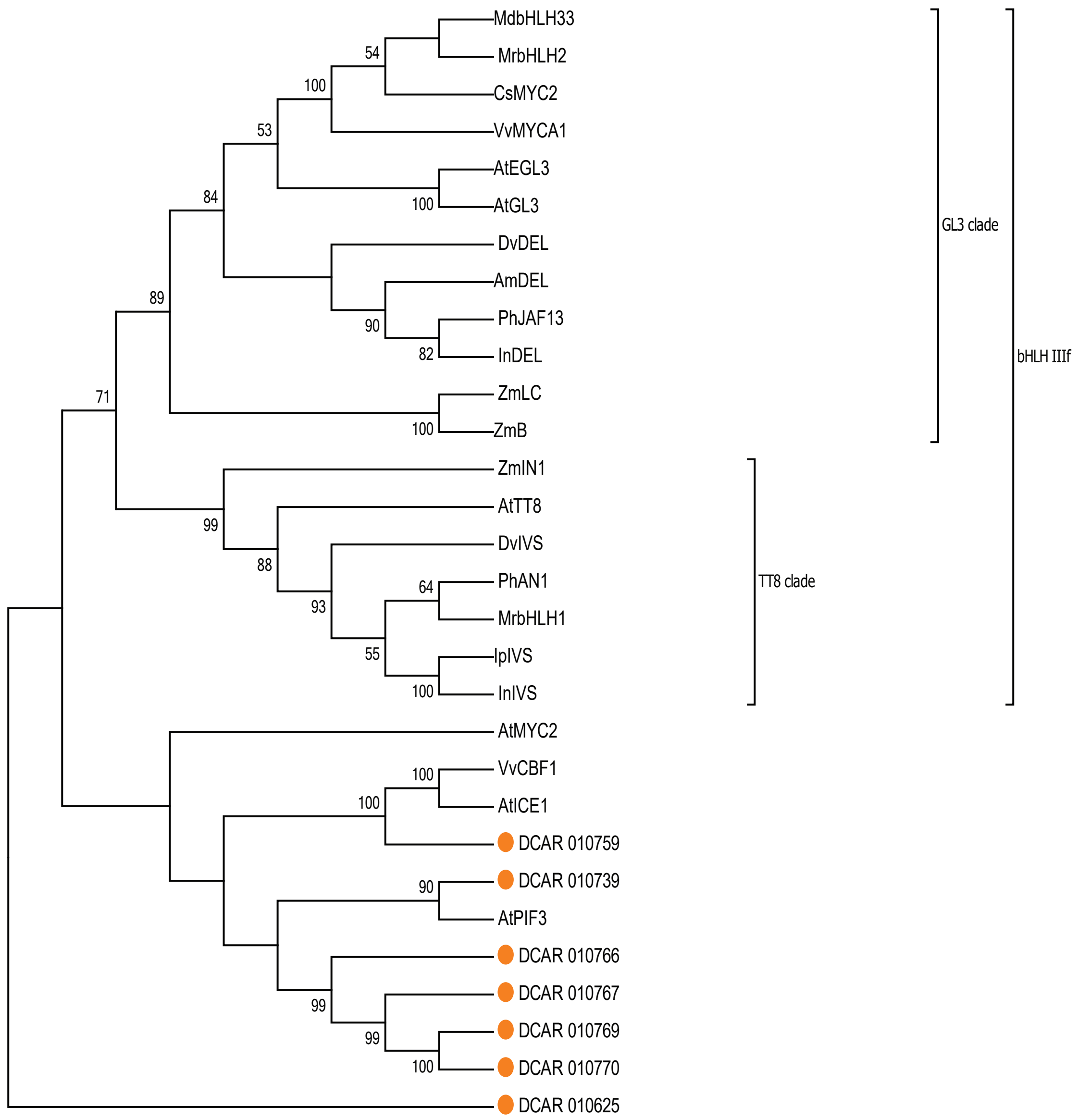

Supplementary Figure S5. Phylogenetic relationships among carrot bHLH genes located in the RTPE-Q1/P3 regions and bHLH genes from other species. Anthocyanin-related bHLH belong to the bHLH IIIf group. The tree was constructed with MEGA 7 software, using a neighbor-joining phylogeny testing, with 1,000 bootstrap replicates. The gene IDs and accession numbers of these proteins (or translated products) are as follows in the GenBank database: AtTT8, CAC14865.1; AtGL3, NP_680372; AtEGL3, NP_176552; MdbHLH33, ABB84474.1; PhJAF13, AAC39455; IpIVS, BAD18982.1; VvMYCA1, NP_001267954.1; CsMYC2, ABR68793.1; PhAN1, AAG25927; AmDEL, AAA32663; DvIVS, BAJ33515; DvDEL, BAJ33516; InDEL, BAE94393; ZmB, AGO65322.1; ZmLC, NP_001105339.1; InIVS, BAE94394; ZmIN1, AAB03841; MrbHLH1, JX629461; MrbHLH2, JX629462; AtMYC2, NP_174541.1; AtICE1, NM_113586.3; VvCBF1, AFI49627.1; AtPIF3, NM_179295.2; AtBEE1, AY138253.1.

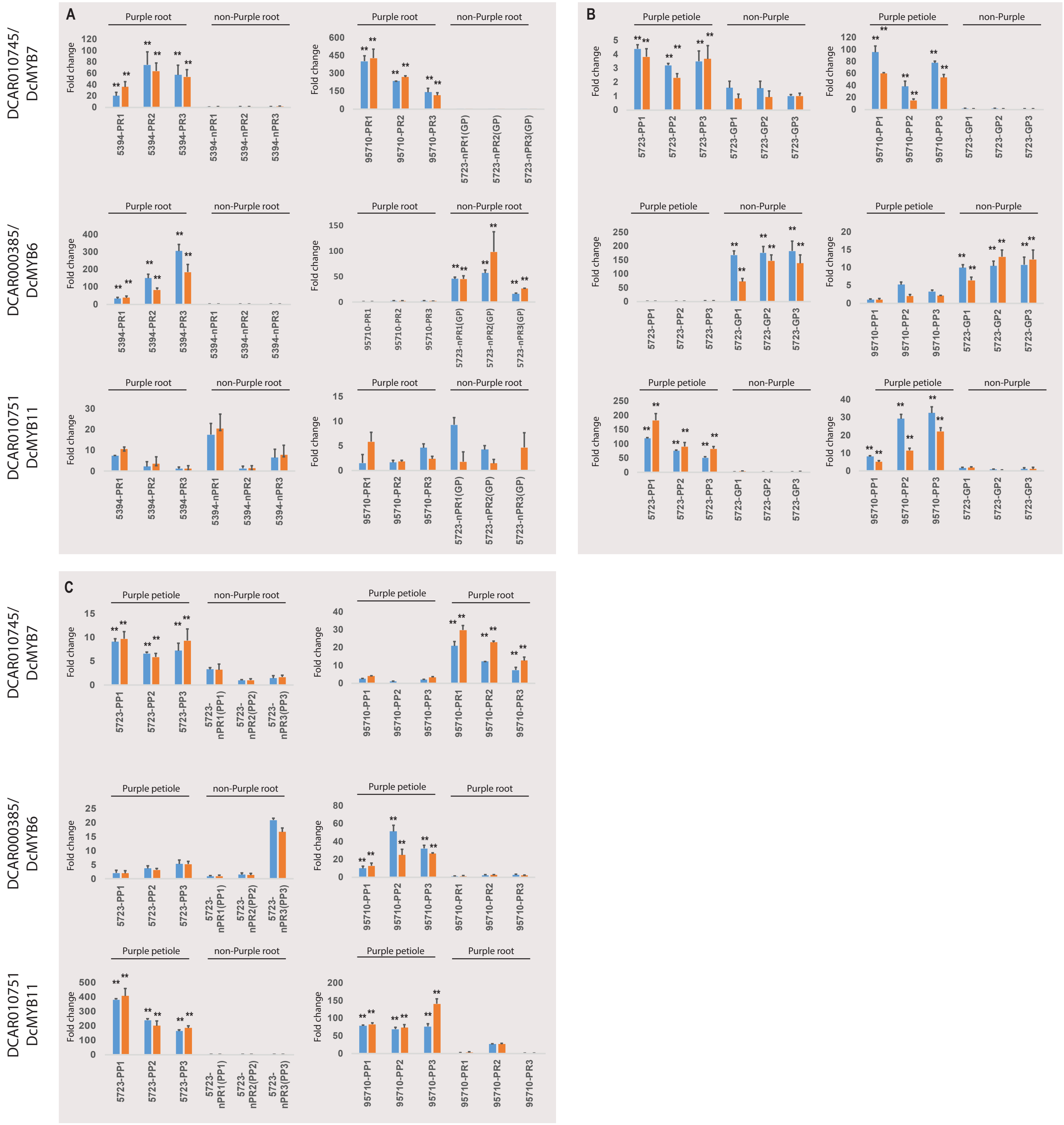

Supplementary Figure S6. Gene expression analysis of candidate genes for RTPE-Q1 and P3 by reverse transcription quantitative PCR (RT-qPCR) analysis. Transcript levels of DCAR000385 (DcMYB6), DCAR010145 (DcMYB7), and DCAR010751 (DcMYB11), were calculated relative to the expression level of the endogenous carrot genes EF-1 (blue bar) and ACTIN (orange bar). Each bar represents the average fold change expression level in each biological replicate. Error bars represent the SD of three technical replicates. To compare the expression differences of the genes in each comparison, statistical analyses were performed based on Tukey's HSD-test following one-way ANOVA". ** P <0.01; * P <0.05. Significant levels are reported only for those comparisons in which all three biological replicates were significantly different. A: purple root vs non-purple root comparisons; B: Purple petioles vs non-purple petiole comparisons; C: purple petiole vs non-purple root comparisons (same plants) and purple petiole vs purple root comparison (same plants).
